# Edge effects and vertical stratification of aerial insectivorous bats across the interface of primary-secondary Amazonian rainforest

**DOI:** 10.1371/journal.pone.0274637

**Published:** 2022-09-23

**Authors:** Natalie Yoh, James A. Clarke, Adrià López-Baucells, Maria Mas, Paulo E. D. Bobrowiec, Ricardo Rocha, Christoph F. J. Meyer

**Affiliations:** 1 School of Science, Engineering and Environment, University of Salford, Salford, United Kingdom; 2 Durrell Institute of Conservation and Ecology, University of Kent, Canterbury, United Kingdom; 3 Statistical Ecology at Kent, National Centre for Statistical Ecology, School of Mathematics Statistics and Actuarial Science, University of Kent, Canterbury, United Kingdom; 4 Natural Sciences Museum of Granollers, Granollers, Spain; 5 Biological Dynamics of Forest Fragments Project, National Institute for Amazonian Research and Smithsonian Tropical Research Institute, Manaus, Brazil; 6 CIBIO, Centro de Investigação em Biodiversidade e Recursos Genéticos, InBIO Laboratório Associado, Campus de Vairão, Universidade do Porto, Vairão, Portugal; 7 CIBIO, Centro de Investigação em Biodiversidade e Recursos Genéticos, InBIO Laboratório Associado, Instituto Superior de Agronomia, Universidade de Lisboa, Lisboa, Portugal; 8 Department of Biology, University of Oxford, Oxford, United Kingdom; Irstea, FRANCE

## Abstract

Edge effects, abiotic and biotic changes associated with habitat boundaries, are key drivers of community change in fragmented landscapes. Their influence is heavily modulated by matrix composition. With over half of the world’s tropical forests predicted to become forest edge by the end of the century, it is paramount that conservationists gain a better understanding of how tropical biota is impacted by edge gradients. Bats comprise a large fraction of tropical mammalian fauna and are demonstrably sensitive to habitat modification. Yet, knowledge about how bat assemblages are affected by edge effects remains scarce. Capitalizing on a whole-ecosystem manipulation in the Central Amazon, the aims of this study were to i) assess the consequences of edge effects for twelve aerial insectivorous bat species across the interface of primary and secondary forest, and ii) investigate if the activity levels of these species differed between the understory and canopy and if they were modulated by distance from the edge. Acoustic surveys were conducted along four 2-km transects, each traversing equal parts of primary and ca. 30-year-old secondary forest. Five models were used to assess the changes in the relative activity of forest specialists (three species), flexible forest foragers (three species), and edge foragers (six species). Modelling results revealed limited evidence of edge effects, except for forest specialists in the understory. No significant differences in activity were found between the secondary or primary forest but almost all species exhibited pronounced vertical stratification. Previously defined bat guilds appear to hold here as our study highlights that forest bats are more edge-sensitive than edge foraging bats. The absence of pronounced edge effects and the comparable activity levels between primary and old secondary forests indicates that old secondary forest can help ameliorate the consequences of fragmentation on tropical aerial insectivorous bats.

## Introduction

Deforestation and fragmentation of tropical forests continue to be major contributors to global biodiversity loss [1]. The Brazilian Amazon currently hosts over 10,000 plant species and is a global hotspot for terrestrial vertebrate diversity [2,3]. Declines in Amazonian deforestation over the last two decades provided some with optimism for Brazil’s commitment to conservation. However, in 2020 the Brazilian Amazon experienced the highest deforestation rates for the last decade [4]. This was largely driven by the dismantling of environmental regulations and enforcement capacity, compounded by political and economic uncertainty left by the wake of the COVID-19 tragedy in Brazil [4–7]. Such deforestation has massive implications for global biodiversity, as well as global carbon emissions [5,8,9].

Deforestation creates a patchwork of isolated forest fragments across modified landscapes. The interface between these artificially created fragments and the matrix (e.g., pasture or agricultural land) is subjected to edge effects [10]. Edge effects, the changes in abiotic conditions and biotic interactions at the boundary between two contrasting habitats, are strong determinants of ecological processes in humanized landscapes [1,11]. As edge conditions exceed the variability typically associated with habitat interiors, environmental deterioration often decreases the habitat suitability for the assemblages it previously supported [8,11–15]. Approximately 70% of remaining global forests are within 1 km of the edge [1] and 85% of 1,673 vertebrate populations are already affected by edge effects [11], with edge area globally increasing from 27% to 37% over the last decade [16]. By 2100, half of tropical forest is predicted to become forest edge [16]. In the Brazilian Amazon, at least 35,000–50,000 km of new edge is created annually [17].

Despite being one of the most well-studied ecological phenomena of the last century [18], edge effects are still not well understood due to their diversity and complexity. Two components of edge influence, edge extent and edge magnitude, can be distinguished. The extent of edge effects is defined as the distance over which changes in natural conditions that are associated with habitat boundaries penetrate habitat interiors, whereas magnitude is the relative strength of an edge effect [19]. Both metrics are highly taxon- and context-specific, and the range of edge-effect extent is widely debated. Most edge effects have been documented to occur between 100–300 m from the edge (e.g., changes to canopy height and understory bird densities; [15,19,20]). However, other studies estimate they may extend 1–10 km into forest interiors (e.g., shifts in carnivore abundance; [21–25]). Matrix composition is known to significantly affect both the extent and magnitude of edge effects, with low-contrast matrices (e.g., secondary forest in advanced regeneration) increasing connectivity between remnant forest patches and reducing the gradient of microclimatic change [12,26–28]. Therefore, forest regeneration can lead to ‘edge sealing’ or ‘edge softening’ [26], as the disturbed, secondary forest can provide habitat for primary forest (forest relatively undisturbed by human activities) specialists. Many tropical studies fail to consider source-sink dynamics between populations in primary forest and the matrix [29]. This is the process whereby species can persist in the secondary forest (a “sink” habitat) so long as there is continual immigration from primary forest (a “source” habitat). Without such proximity to the source habitat, populations in the sink habitat would begin to decline [29]. As such, studies comparing species responses across a habitat boundary should consider the habitats on either side as interactive and not as independent units.

There have been over 405 reforestation projects across the Brazilian Amazon since 1950, and vast areas of abandoned pastureland are now under natural forest regeneration [9]. Between 1986 and 2018, over 260,000 km^2^ of secondary forest has regenerated in the Brazilian Amazon which equates to almost 60% of the area of old-growth forest which was lost between 1988 and 2019 [4,30]. Secondary forests are increasingly recognized for improving species’ persistence in tropical human-modified landscapes [31]. As secondary forests mature, they reduce the gradient of structural differences between the matrix and the primary forest [28,31,32]. This helps to mitigate the impact of edge effects in primary forest and increase habitat suitability across the landscape [32,33]. Whilst secondary forests are no substitute for old-growth forests, they typically support around 57% of the diversity of primary forests [31], even after only 14–19 years of regeneration [34]. For indicator species, such as dung beetles and birds, there is evidence to suggest secondary forests can support the equivalent diversity of primary forests within 15 to 30 years, respectively [35]. As secondary regrowth continues to mature, it has been shown to support more forest specialist species, including bats [32,35–38].

The Amazon supports over 200 bat species that perform important ecological roles in tropical forests, such as pollination, seed dispersal and insect suppression [39,40]. Few studies to date have investigated how tropical bats respond to edge effects and existing studies have focused predominantly on phyllostomids, the ecologically most diverse Neotropical bat family, as these species can be reliably sampled using mist nets [e.g., 12,22,41]. These studies suggest bats may be affected by edge effects up to 3 km from the habitat boundary [22], with most studies indicating species richness declines at the forest edge, whereas the abundance of several dominant generalist species increases [13,42]. Aerial insectivores, which represent a large fraction of Amazonian bat diversity [43], have so far been overlooked. There have also been limited studies investigating how fragmentation and edge effects may affect bats differently between forest strata [but see 43–45]. It is widely accepted that there are differences in both bat diversity and abundances between the canopy and understory in the Amazon [46,47]. However, due to sampling logistics, it is often difficult to incorporate canopy sampling into mist-netting surveys. Alternatively, acoustic monitoring enables us to include aerial insectivores in such studies and provides an effective method for cross-strata comparisons, thereby providing a more comprehensive understanding of how the wider bat community may be impacted by edge effects.

Working within an experimentally fragmented landscape with low fragment-matrix contrast, the overarching goal of this study was to assess edge influence, both in terms of extent and magnitude, on Amazonian aerial insectivorous bats. Specifically, we evaluated how bat activity varied along a habitat gradient of increasing distance from the habitat boundary in both secondary and primary forest. We assessed how this response in activity varied along this gradient between the understory and canopy. These comparisons were conducted for common species/sonotypes and three functional guilds. We hypothesized that forest specialist activity would exhibit a negative edge effect response in both habitats, whereas we expected to see a positive or null response for flexible forest foragers and edge foragers. Furthermore, we anticipated that responses to edge effects differ between the understory and canopy, with a greater extent and magnitude being observed in the canopy.

## Materials and methods

This research was conducted under ICMBio (Instituto Chico Mendes de Conservação da Biodiversidade) permit (26877–3).

### Study sites

**Fig 1:**
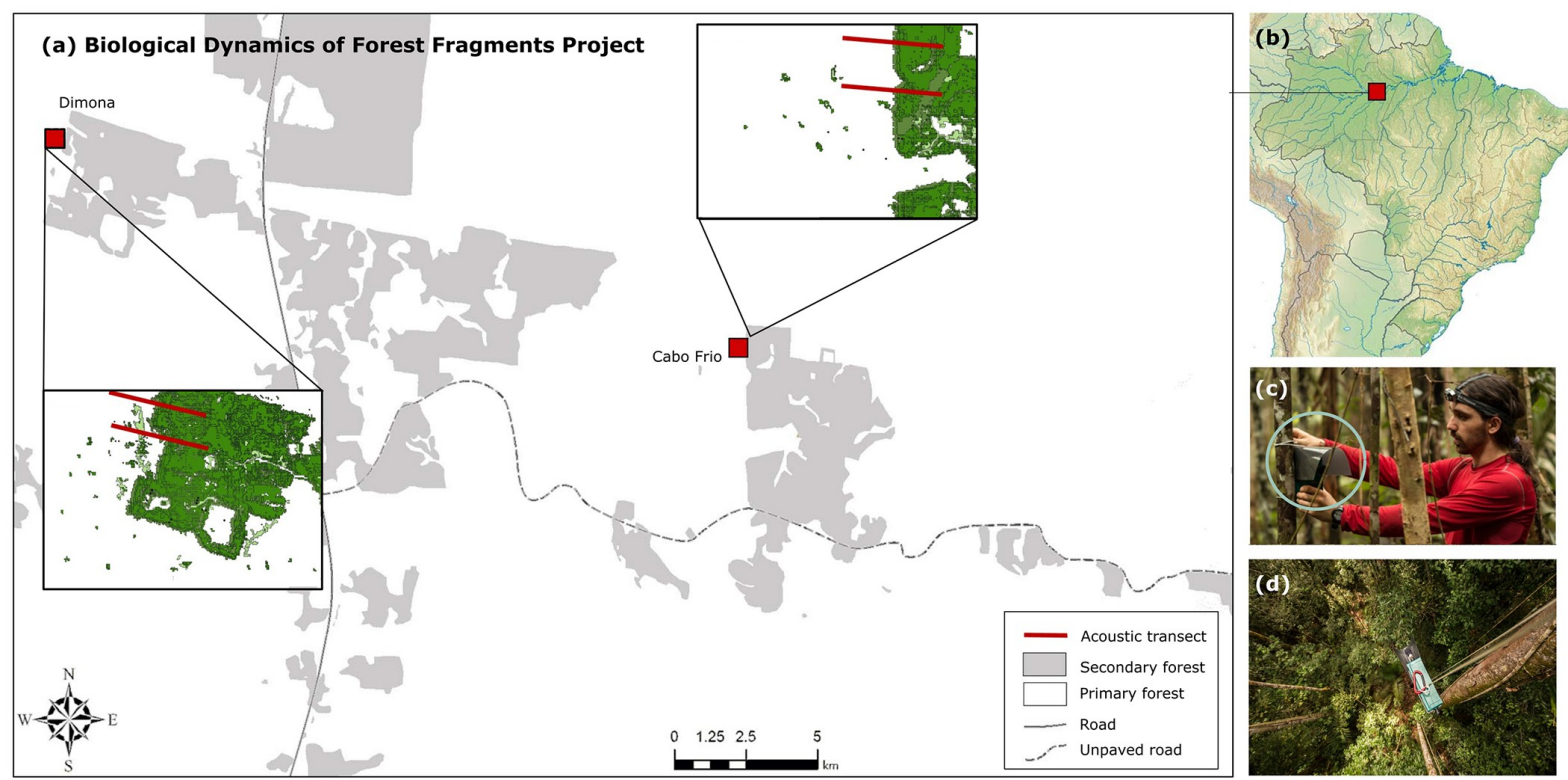
Location of the primary-secondary forest transects at the Biological Dynamics of Forest Fragments Project, Central Amazon, Brazil. (a) Transect location within the BDFFP is presented in the inserts, where primary forest is denoted in white and secondary forest is presented in green. (b) Location of the BDFFP in Brazil. Photographs show the plastic dividers used to ensure acoustic recordings from the understory (c) and canopy samples (d) were independent.

Our study was conducted in the Central Brazilian Amazon, 80 km north of Manaus, at the Biological Dynamics of Forest Fragments Project (BDFFP; 2024’26”S, 59043’40”W; Fig 1). The BDFFP is the world’s most comprehensive, long-term experimental study into the effects of habitat fragmentation across a broad range of taxa [12]. The primary forest is classified as *terra firme* forest, with an average tree diversity of 280 species per hectare [48]. In the early 1980s, a series of primary forest fragments (1, 10, and 100 ha) were experimentally isolated within cattle ranches, separated 80–650 m from continuous forest. However, forest regeneration quickly occurred after the ranches were abandoned 5–10 years later due to economic unviability [49,50]. Regrowth forest was dominated by *Vismia* spp., in areas that were cleared and burned, or *Cecropia* spp., in areas that were cleared without fire [51]. The understory is dominated by palms [50] and is characterized by an average canopy height of 23 m [50]. The secondary forest at the time of the study was classified as ‘old secondary forest’ using the age classes proposed by Powell et al. [38] (27–31 years old with a mean canopy height ≥ 19 m). A small strip has been periodically cleared to ensure fragment isolation, most recently between late 2013 and early 2014 [52]. Average annual rainfall ranges between 2.3–2.5 m, with large interannual variation (1.9–3.5 m). The wet season occurs between November and June (monthly rainfall > 250 mm) and the dry season occurs between July and October (monthly rainfall < 100 mm). The average temperature is between 26–30°C and the study area is characterized by low-lying topography (80–160 m elevation [32,53]).

## Acoustic sampling

Two static detectors (SM2Bat+, Wildlife Acoustics) with omnidirectional microphones (SMX-US Ultrasonic Microphone) were placed in the understory and canopy of 164 sample points. These sample points were spaced 50 m apart along four 2-km transects. Transects were located across two spatially independent sites to reduce site bias (Fig 1). Each transect extended through 1 km of secondary forest and then continued 1 km into the neighboring primary forest. Surveys were conducted in the dry season of 2013 and the wet season of 2014 to minimize seasonal bias, equating to eight transect visits in total. As bats are known to favour established flyways for commuting [54], each transect was established specifically for this study.

As Amazonian bats are known to exhibit vertical stratification [45,55], we recorded bats separately using two detectors simultaneously, with one detector in the understory and one in the canopy. For this study, the understory was defined as extending from the ground to a height of approximately 10 m and the canopy was defined as approximately 30 m from ground level. To ensure the understory and canopy samples could be considered independent, plastic dividers were attached to the detectors to create discrete directional microphones (Fig 1C; [56]). Additionally, sample points were manually rotated so that actively recording detectors were always 250 m apart. Each active detector was programmed to record for 12 hours (18:00–06:00) for three consecutive nights, amounting to 11,808 recording hours. Detectors recorded at 384 kHz sampling rate in full spectrum with 16-bit resolution. The high pass filter was set at 12 kHz (fs/32), with a trigger level of 18SNR. Recordings were split into five-second fragments with at least two distinguishable pulses to define a bat pass which was used as the surrogate measure of bat activity [57].

### Call classification to species/sonotype

We used a combination of manual and automatic methods to classify calls to species or sonotype (a group of species with similar calls). We tested which species could be reliably classified using automated methods by first manually processing a subset of calls (all calls recorded in the understory) and then comparing the results against those generated using the classifier for Amazonian bats developed by López-Baucells et al. [58]. To improve the performance of the classifier, we included additional reference calls into the classifier training dataset (S1 Table) following the methodology of López-Baucells et al. [58]. We compared the difference between manual identification (45,554 bat passes) and automatic identification (41,702 bat passes) of the understory data using non-paired Wilcoxon Signed-Rank tests to confirm the reliability of the automatic classifications. Overall, the automatic classifier generated comparable results to calls identified manually (W = 123,260, *p* = 0.87). However, to increase consistency and robustness for the edge-effect analysis, we only included the calls for those 12 species where there was no difference between manual identification and automatic identification. See supporting information for full classifier performance results.

### Statistical analysis

**Fig 2:**
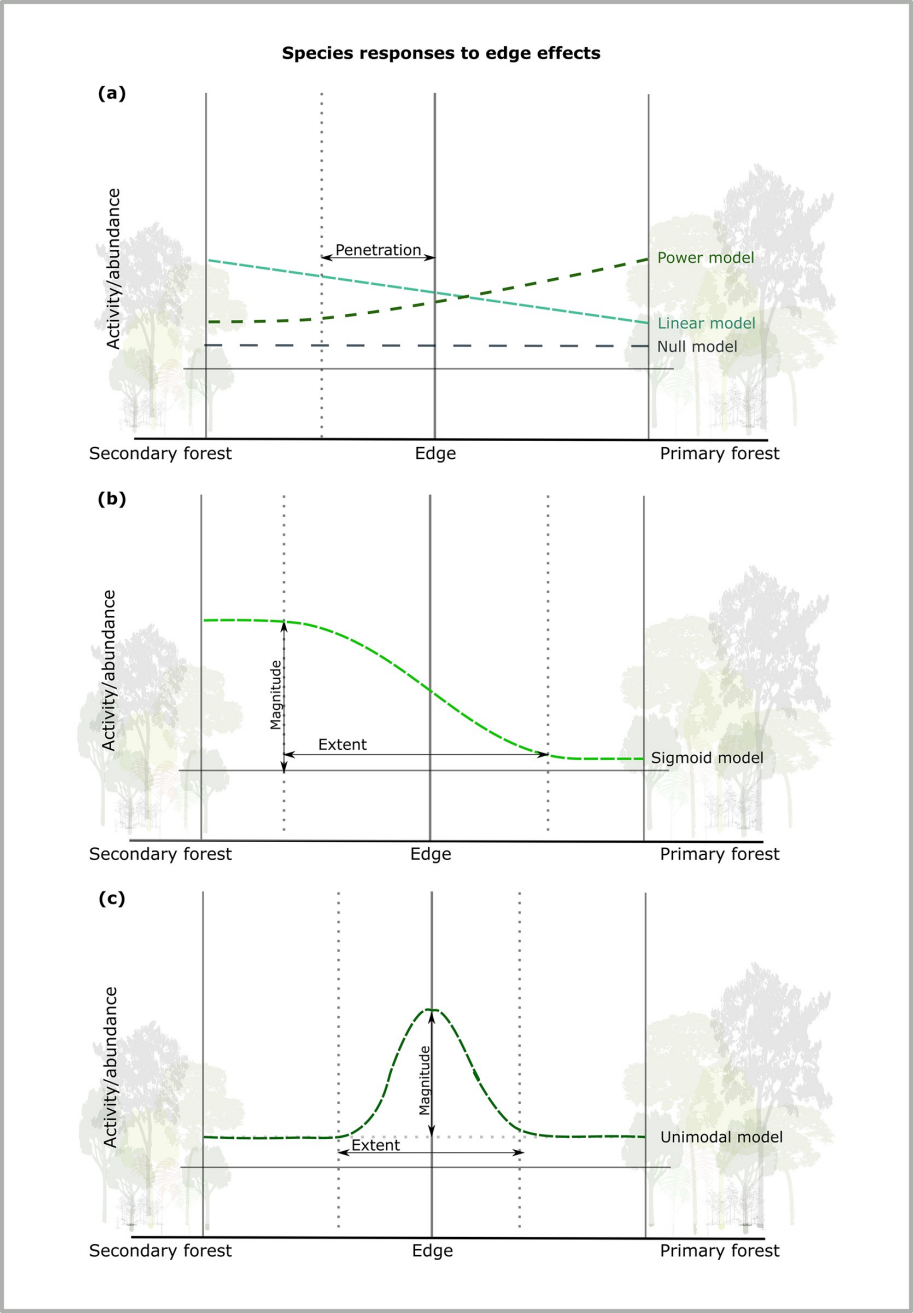
Visual representation of the five models proposed by Ewers & Didham [19] to delineate species’ theoretical responses to edge effects. (a) For the null, linear, and power models it is not possible to calculate extent or magnitude as there is either no response present or the response exceeds the sampling area; (b) in the sigmoid model, species exhibit a negative response to edge effects and asymptotes are reached in each habitat; and (c) in the unimodal model, species demonstrate a preference for edge habitat. Note, we have illustrated here a hypothetical preference for secondary forest using the linear and sigmoid model and a preference for primary forest using the power model. However, each of these models can be used to demonstrate a preference for either habitat.

The 12 species we selected were assigned to guild depending on their ecological requirements and family [59,60]. Species/sonotypes that were considered forest specialists included *Eptesicus brasiliensis*, *Furipterus horrens*, and *Myotis riparius*. *Pteronotus* spp. also typically inhabit forest areas. However, in our study, we have defined them as ‘flexible forest’ species as they are known to commonly exploit other habitats, such as forest edges, as well as hunt in highly cluttered spaces [61,62]. The final guild consisted of six ‘edge’ species/sonotypes which typically forage along forest edges or in forest gaps. This included *Cormura brevirostris*, *Centronycteris maximiliani/centralis*, *Peropteryx kappleri*, *P*. *macrotis*, *Saccopteryx bilineata*, and *S*. *leptura*. By grouping species, we were able to assess guild-level responses to edge effects. Continuous response functions, as described in Ewers and Didham [19], were used to identify edge effects across the primary and secondary forest interface (Fig 2).

The five models can be used to describe mean bat activity per guild, η, at a certain distance (D) from the edge, and these models are as follows:

### (1) Null model


$$\eta_{D}=\bar{\eta }+\epsilon$$ (1)

With ϵ denoting the error term and $\bar{\eta }$ mean bat activity across all distances from the edge. This model describes a scenario in which no discernible edge effect can be detected using the data (i.e., generalist activity).

### (2) Linear model


$$\eta_{D}=\beta_{0}+\beta_{1}D+\epsilon$$ (2)

This model describes a simple linear gradient in mean bat activity for a particular guild across the edge. *β*_0_ and *β*_1_ denote constants and D the distance from the habitat edge.

### (3) Power model


$$\eta_{D}=\beta_{0}e^{\beta_{1}D}+\epsilon$$ (3)

This model describes a scenario in which there is an asymptote on one side of the edge.

### (4) Sigmoid model


$$\eta_{D}=\beta_{0}+\frac{\beta_{1}-\beta_{0}}{1+e^{(\beta_{2}-D)\beta_{3}}}+\epsilon$$ (4)

This model describes a scenario in which there is an asymptote on each side of the edge, with *β*_2_ and *β*_3_ as constants. This represents groups in which there is a discrete change in activity from one habitat to the next.

### (5) Unimodal model


$$\eta_{D}=\beta_{0}+\frac{\beta_{1}-\beta_{0}}{1+e^{(\beta_{2}-D+\beta_{4}D^{2})\beta_{3}}}+\epsilon$$ (5)

This model describes a situation similar to the sigmoid model, but with a clear peak in the response at the edge (i.e. groups with a preference for habitat edges). This is described through the inclusion of the constant *β*_4_.

The canopy and understory data for each guild were analysed separately as we expected that the model of best fit would depend on forest stratum. Average activity was log-transformed to ensure normality assumptions were met. Non-linear models were fit using the “nlsLM” function from the R package “minpack.lm” [63]. Once each model was fitted, we compared them using the second-order Akaike Information Criterion (AICc) to determine the model of best fit whilst correcting for small sample sizes [64]. An advantage of using these models is the ability to calculate the magnitude and extent of the edge effects for Eqs 4 and 5, if they were the best-fit models. Full model parameters are available in S2 Table.

We then applied generalized linear mixed effect models (GLMMs) with negative binomial distributions to determine if bat activity varied with distance from the edge or between strata. Only non-correlated variables were included in the models to avoid collinearity (r_s_ < 0.5). Activity data was not log-transformed in the GLMMs [65]. The final fixed covariates were *Strata* (“understory” vs. “canopy,” categorical with two levels), *ForestType* (categorical with three levels) and *Distance* (continuous). We included *Transect* as a random intercept, to incorporate the dependency among observations of the same transects, as well as *Season*, to account for any seasonal variation in activity. All covariates were centred and standardized before analysis [66]. We fit the models using the package “glmmADMB” [67] (S3 Table). The top three models were determined based on their AICc values. We then undertook likelihood ratio tests to determine which covariates from these models were statistically significant (S4 Table). The best-fit model included all covariates identified as statistically significant from the likelihood ratio tests. This analysis was repeated for each guild and species/sonotype.

### Results

In total 252,912 bat passes were automatically identified to 12 aerial insectivorous species or sonotypes. This included species from four families: two *Vespertilionidae* species/sonotypes, six *Emballonuridae* species/sonotypes, three *Mormoopidae* species and one species of *Furipteridae* (Table 1). Three species/sonotypes were not included in the edge effect analysis. This includes *Emballonuridae* spp. (n = 8,205) and *Pteronotus personatus* (n = 459), which had insufficient bat passes manually identified in the understory to test for agreement between the manual and automatic identification methods, and *Molossidae* spp. (n = 9,236) as we found the automatic classification for this sonotype was significantly different from manual identification, suggesting incorrect classifications (S1 Table). Finally, three bat passes were manually identified as *Thyroptera tricolor* in the understory but this species is not specified in the automatic classifier and therefore was excluded.

**Table 1:** Total number of bat passes per species/sonotype in both the understory and canopy of secondary forest, forest edge, and primary forest.

			Understory			Canopy			Total
			Secondary	Edge	Primary	Secondary	Edge	Primary	
**Forest specialists**									
	*Eptesicus brasiliensis*		99	2	29	589	21	945	1,685
	*Furipterus horrens*		25	0	10	27	1	13	76
	*Myotis riparius*		489	16	148	2,629	105	1,390	4,777
**Flexible forest foragers**									
	*Pteronotus gymnonotus*		164	5	72	336	17	143	737
	*Pteronotus alitonus*		5,573	278	4,444	5,579	159	4,020	20,053
	*Pteronotus* cf. *rubiginosus*		5,773	136	2,660	1,699	37	959	11,264
**Edge foragers**									
	*Cormura brevirostris*		188	4	259	1,781	50	3,317	5,599
	*Centronycteris maximiliani/centralis*		10,838	7	4,370	51,742	4,352	50,651	121,960
	*Peropteryx kappleri*		82	1	22	3,196	151	3,717	7,169
	*Peropteryx macrotis*		337	3	195	6,923	1,174	5,238	13,870
	*Saccopteryx bilineata*		604	4	2,416	6,089	836	30,319	40,268
	*Saccopteryx leptura*		271	0	651	7,231	957	16,344	25,454
**Excluded from analysis**									
	*Emballonuridae* spp.		177	10	182	2,629	982	4,225	8,205
	*Molossidae* spp.		438	44	733	5,195	159	2,667	9,236
	*Pteronotus personatus*		46	0	17	222	5	169	459
	*Rhynchonycteris naso*		0	0	0	8	0	2	10
	Total		25,104	510	16,208	95,875	9,006	124,119	270,822

These values represent bat passes as determined by the automatic classifier. Data for *Thyroptera tricolor* not given as this species was only identified manually and is not included in the classifier.

**Fig 3:**
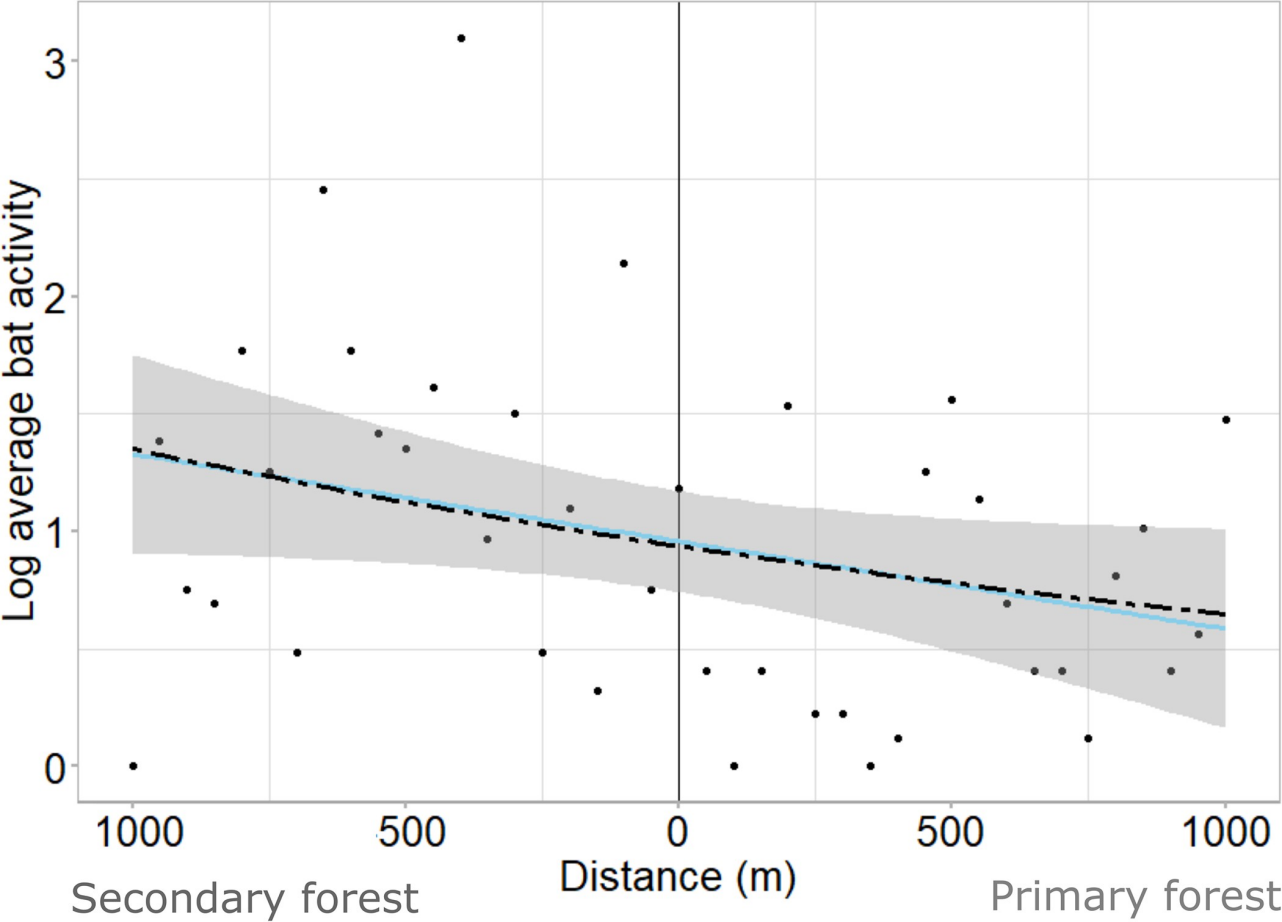
Edge effect model fit for forest specialists in the understory. Log forest specialist activity (bat passes) per 50 m sample point averaged across all transects with corresponding lines of best fit. Activity increases from the edge in the secondary forest and decreases from the edge in the primary forest. Dark blue dash–power model, light blue–linear model. Standard error provided for the linear model. Model parameters listed in S2 Table.

The null model provided the model of best fit for forest specialists in the canopy which indicates that there was no edge effect on mean activity in this stratum (Table 2). The linear and power models provided the best fit for forest specialists in the understory. There was little variation between the linear and power model fit (Fig 3). Despite considerable variation in the data, both models showed that activity increased from the interior in primary forest towards the edge and into the secondary forest, whereby activity peaked in secondary forest farthest from the forest edge, therefore indicating a preference for secondary forest (Fig 3).

**Table 2:** Comparison of model fit using Ewers and Didham’s (2006) edge effect models.

Guild	Habitat	Model	AICc	
Forest specialists				
	Canopy			
		**Null**	**116.357**	*
		Linear	118.245	
		Power	118.258	
		Sigmoid	121.775	
		Unimodal	125.423	
	Understory			
		Null	91.427	
		**Linear**	**89.507**	*
		**Power**	**89.722**	*
		Sigmoid	90.100	
		Unimodal	98.100	
Flexible forest foragers				
	Canopy			
		**Null**	**114.132**	*
		Linear	116.003	
		Power	115.974	
		Sigmoid	116.072	
		Unimodal	119.413	
	Understory			
		**Null**	**127.412**	*
		Linear	129.054	
		Power	129.066	
		Sigmoid	131.469	
		Unimodal	137.224	
Edge foragers				
	Canopy			
		**Null**	**148.845**	*
		Linear	149.609	
		Power	149.654	
		Sigmoid	155.166	
		Unimodal	157.421	
	Understory			
		**Null**	**162.503**	*
		Linear	164.652	
		Power	164.655	
		Sigmoid	169.718	
		Unimodal	171.593	

Results are provided for each of the three guilds in both the understory and canopy. Bold*—model/(s) of best fit.

For both flexible forest foragers and edge foragers, we found the null model provided the best fit for both the canopy and understory. This indicates there is no evidence that edge effects were affecting either of these guilds (Table 2). In contrast to our hypothesis, the unimodal models provided the poorest fit for edge foragers. No calculations were possible for edge extent or magnitude as no guild demonstrated a relevant edge effect response (e.g., sigmoid or unimodal), and it is not recommended to infer magnitude or extent from the power model [19].

Distance from the edge did not explain edge forager activity or forest specialist activity based on GLMMs (Table 3, S3 and S4 Tables). However, compared to the habitat boundary, flexible forest forager activity was significantly higher with increasing distance from the edge (Table 3). There was no difference in response between primary and secondary forest and the forest edge for any guild (Table 3). We also observed no significant differences in bat activity between the primary and secondary forest or edge for any species/sonotype (Table 3). Only one species demonstrated a significant response to distance from the edge, *Peropteryx macrotis*, which had greater activity closer to the edge.

**Table 3:** Summary of the best-fit generalized linear mixed effect models for each bat guild and species/sonotype.

		Estimate	SE	z	*p-*value	
Forest specialists						
	Intercept	3.595	0.446	8.05	8.1^e-16^	***
	Understory	-1.609	0.144	-11.17	< 2^e-16^	***
	Primary forest	0.530	0.426	1.24	0.210	
	Secondary forest	-0.036	0.427	-0.08	0.930	
Flexible forest foragers						
	Intercept	4.287	0.464	9.24	< 2^e-16^	***
	Understory	0.449	0.140	3.26	0.001	**
	Primary forest	0.170	0.416	0.41	0.683	
	Secondary forest	-0.193	0.426	-0.45	0.650	
	*Distance*	1.233^e-03^	2.66^e-04^	1.97	0.048	*
Edge foragers						
	Intercept	6.504	0.607	10.72	< 2^e-16^	***
	Understory	-2.177	0.181	-12.06	< 2^e-16^	***
	Primary forest	0.552	0.525	1.05	0.294	
	Secondary forest	0.896	0.538	1.66	0.096	
*Eptesicus brasiliensis*						
	Intercept	2.797	0.102	27.43	< 2^e-16^	***
	Understory	-1.350	0.202	-6.67	2.6^e-11^	***
*Furipterus horrens*						
	Intercept	1.183	0.208	5.69	1.2^e-08^	
*Myotis riparius*						
	Intercept	3.393	0.473	7.18	7^e-13^	***
	Understory	-1.397	0.162	-8.64	< 2^e-16^	***
	Primary forest	0.469	0.454	1.03	0.300	
	Secondary forest	-0.182	0.457	-0.40	0.690	
*Pteronotus gymnonotus*						
	Intercept	1.670	0.327	5.11	3.2^e-07^	***
	Understory	-0.321	0.127	-2.53	0.011	*
	Primary forest	0.390	0.331	1.18	0.239	
	Secondary forest	-0.034	0.336	-0.10	0.919	
*Pteronotus alitonus*						
	Intercept	4.340	0.303	14.30	< 2^e-16^	***
*Pteronotus* cf. *rubiginosus*						
	Intercept	3.456	0.327	10.56	< 2^e-16^	***
	Understory	0.821	0.182	4.51	6.5^e-06^	***
*Cormura brevirostris*						
	Intercept	3.641	0.294	12.37	< 2^e-16^	***
	Understory	-1.548	0.164	-9.41	< 2^e-16^	***
*Centronycteris maximiliani/ centralis*						
	Intercept	6.558	0.408	16.08	< 2^e-16^	***
	Understory	-1.743	0.214	-8.15	3.7^e-16^	***
*Peropteryx kappleri*						
	Intercept	4.112	0.150	27.40	< 2^e-16^	***
	Understory	-2.632	0.257	-10.20	< 2^e-16^	***
*Peropteryx macrotis*						
	Intercept	4.967	0.334	14.85	< 2^e-16^	***
	Understory	-2.107	0.194	-10.88	< 2^e-16^	***
	*Distance*	-0.001	3.25^e-03^	-4.19	2.8^e-05^	***
*Saccopteryx bilineata*						
	Intercept	5.154	0.618	8.34	< 2^e-16^	***
	Understory	-1.858	0.204	-9.11	< 2^e-16^	***
	Primary forest	-0.452	0.586	0.77	0.440	
	Secondary forest	0.928	0.598	1.55	0.120	
*Saccopteryx leptura*						
	Intercept	5.884	0.647	9.10	< 2^e-16^	***
	Understory	-2.577	0.177	-14.59	< 2^e-16^	***
	Primary forest	-1.034	0.648	-1.59	0.110	
	Secondary forest	-0.294	0.648	-0.45	0.650	

See S3 and S4 Tables for complete models.

**Fig 4:**
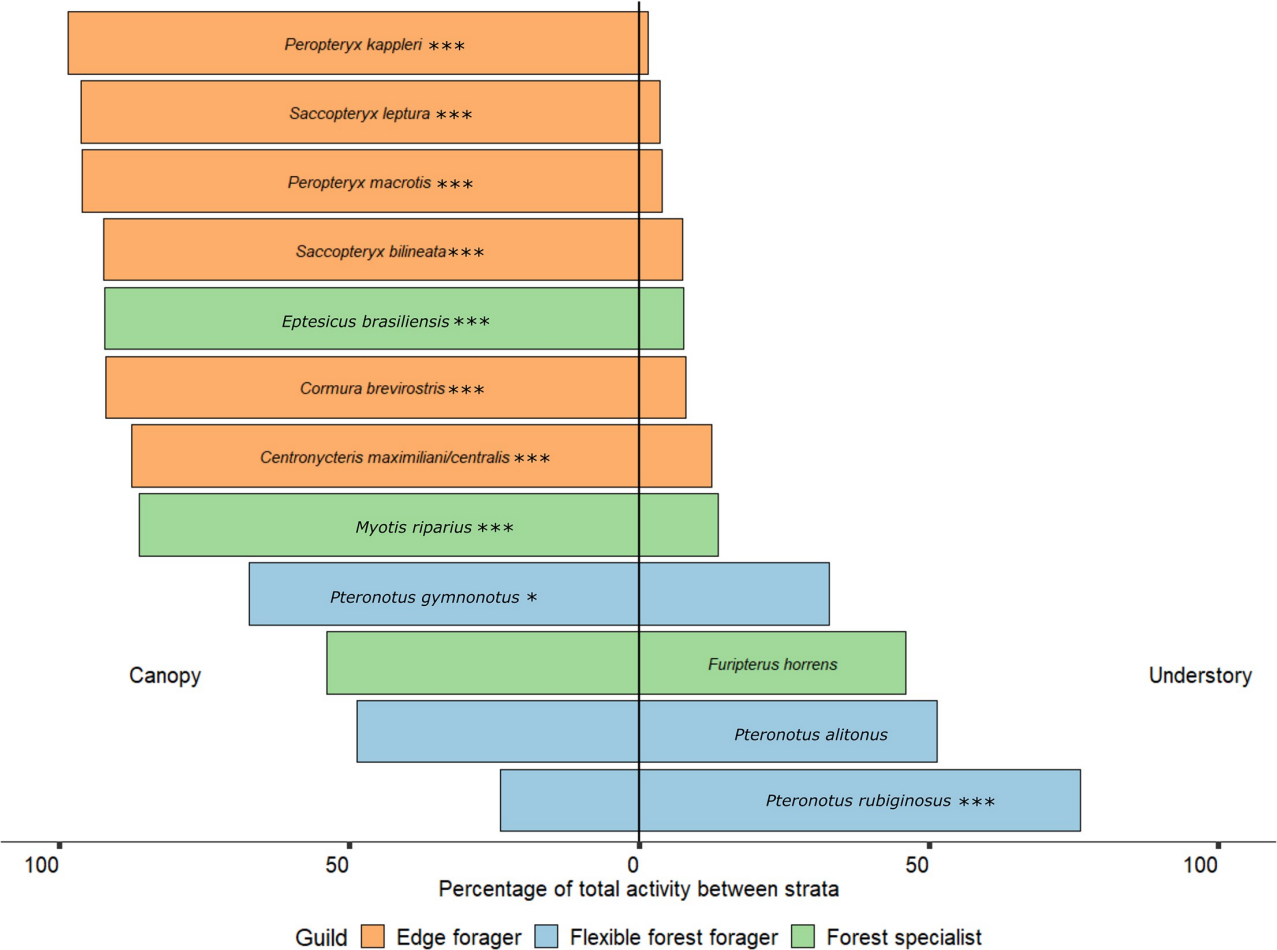
Vertical stratification of twelve Amazonian bat species. Comparison of total bat activity (bat passes) per species/sonotype recorded in the understory and canopy at the Biological Dynamics of Forest Fragments Project. Significance values * < 0.05, *** < 0.001.

We found that stratum was an important predictor for the activity of each guild (Table 3, S3 and S4 Tables). Activity was highest in the canopy for edge foragers and forest specialists, but highest in the understory for flexible forest foragers. We observed that ten of the twelve species were significantly more active in the canopy than the understory (Table 3; Fig 4). Only one species, *Pteronotus* cf. *rubiginosus*, showed a significant preference for the understory.

## Discussion

An expanding body of literature supports the conservation benefits to bats, and multiple other taxonomic groups, associated with the regeneration of secondary forests in fragmented tropical landscapes [32,36,37,68]. By providing evidence of edge sealing, our study supports this by showing that old secondary forest adjacent to primary forest can support comparable activity to primary forest for 12 aerial insectivorous bat species/sonotypes. However, we still found evidence of the impact of edge effects for both forest specialists and flexible forest foragers at the guild level, although results between different statistical approaches were conflicting.

### Guild and species-specific responses

We did not find evidence of edge effects for both flexible forest foragers and edge foragers, as well as forest specialists in the canopy, using Ewers and Didhams’ [19] models. As suggested by Powell et al. [38], the old secondary forest at the BDFFP might have reached the point of recovery where edge effects can no longer be detected for most species/sonotypes. This would be consistent with findings for other taxonomic groups (e.g., dung beetles; [33,36]), suggesting that old secondary forest provides valuable habitat for common aerial insectivores. In contrast, forest specialist activity demonstrated a response to edge effects using Ewers and Didhams’ [19] models, suggesting the secondary forest was not yet sufficiently mature to prevent edge effects penetrating the primary forest. However, different statistical approaches demonstrated conflicting results. Using the GLMM approach, we did find evidence that flexible forest forager activity increased with increasing distance from the edge, whereas no response was detected for forest specialists. Therefore, it is possible these models do not capture the full breadth of response. As such, we advise multiple approaches are used when assessing edge effects.

Whilst old secondary forests at the BDFFP may support several common aerial insectivorous bat species, López-Baucells [61] demonstrated that a complete assemblage-level recovery was not observed after 15 years of forest regrowth. However, recovery rates can vary between bat species and guilds. Even after ~30 years, phyllostomid assemblages in secondary forest may not fully resemble the assemblages within primary forest [32,68]. Trophic level, dispersal ability, and habitat specialization all affect a species’ sensitivity to edge effects [69,70]. Species which are highly dependent on primary forest interiors are more likely to be edge sensitive, to be affected over a larger extent, as well as at greater magnitudes [8,24]. Forest specialist bats typically have low wing loading which gives them the maneuverability to navigate dense forest clutter [71]. Other traits related to their echolocation call design, also facilitate navigating and locating prey in clutter and are poorly suited for more open spaces [71,72]. Compounded, these traits limit their dispersal ability. Fast-flying, more mobile species are less affected by fragmentation as they are more capable of exploiting landscape mosaics [13,61,73]. Whilst we did not observe a significant difference in activity between secondary and primary forest based on the GLMMs, there was evidence forest specialists were to some degree influenced by edge effects in the understory using Ewers and Didhams’ [19] models. However, contrary to our expectations, they exhibited higher activity in the secondary forest. This response may be driven by increased prey availability [74], however we were not able to test this. Increased food availability can lead to an increased abundance of generalist phyllostomids up to 3 km from the forest edge [22]. As the linear model provided the model of best fit for forest specialists in the understory, our results indicate these species may also be impacted by edge effects beyond 2 km.

It is important to note that only four transects in two locations were sampled in this study. Therefore, there may be location-specific factors which have influenced the patterns we observed and the results may not necessarily generalize across the Amazon. Old-growth, continuous forest acts as a source for many species across the BDFFP landscape. Elsewhere in the Amazon, many remaining forest fragments are isolated within a matrix of pasture. As the wider, landscape-scale effects of fragmentation are known to strongly influence edge effects and disrupt source-sink dynamics [11,75], it is likely the magnitude of edge effects in these fragments will be exacerbated. Similarly, whether secondary forest neighbours primary forest is an important determinant of bat abundance and diversity [28]. Many resources may not be available in secondary forest until it matures, e.g., mature/dead trees for roosting. However, more mobile species such as flexible forest foragers and edge foragers may move between habitats to exploit the resources available in each [29]. This could explain why we observe high activity in the secondary forest and would contradict previous findings that suggest that the intermediate disturbance hypothesis does not apply to neotropical bats [28]. Finally, we stress that our study should only be used to draw conclusions about the responses of common species and not to infer how more specialist species are impacted. Nevertheless, our results align with previous studies highlighting that forest species are more edge-sensitive than generalist species [8,24]. As such, primary forest is of irreplaceable value, not only for edge-sensitive phyllostomid bats but also for aerial insectivorous bats [61,76].

### Vertical stratification

Our results support previous findings that tropical bat activity differs between strata, with most species showing a strong preference for the canopy [47,55,77,78]. However, we found different stratum preferences than those previously reported. *Myotis riparius* has previously been shown to prefer the understory in Costa Rica [77] and in French Guiana, where *C*. *maximiliani* also demonstrated a preference for the same stratum [78]. Both were significantly more active in the canopy in our study. *Centronycteris maximiliani* is known to vary its activity in the understory and canopy across the night, with peak canopy activity in the middle of the night [78]. However, this does not account for the differences demonstrated in our study as recordings were collected across the whole night. Both species are relatively small, slow fliers with short call durations (< 6ms) [46,79] which suggests they are well suited to foraging in understory vegetation. Similarly, there has previously been a lack of vertical stratification reported for *Saccopteryx bilineata* and *S*. *leptura* [46,78]. Forest structure is not the only consideration affecting a species’ spatial distribution. Fluctuations in prey availability and moon illumination influence how bats utilize different strata [77,78,80]. Gomes et al. [78] demonstrated how species modulate their stratification preferences across the night to forage opportunistically. However, the scale of the differences we observed in our study (e.g., a seven-fold increase in *C*. *maximiliani* activity between the understory and canopy) suggests a strong affiliation with the canopy. Unlike understory specialists, species that forage in the canopy are considered less vulnerable to the effects of fragmentation, including edge effects [69]. Almost all of the species assessed in this study showed a preference for the canopy. Therefore, our study should not be used to infer how interior, understory specialists will be affected by edge effects.

Whilst we did not detect many direct changes in bat activity in response to edges, the deviation we observed from typical stratum use may reflect the potential for more subtle effects on bat populations. Habitat disturbance, including edge effects, can affect a species’ behaviour, physiology, and other fitness parameters [8,11,81,82]. At least two Amazonian phyllostomids change their habitat preferences to utilize more strata in forest fragments than in continuous forest when locating prey [45]. If edge effects are increasing understory clutter or altering prey distributions, this may have knock-on effects on where bats can forage. This may partially explain why we observe lower understory activity than expected for forest specialists. However, more research is needed to test this hypothesis. Habitat deterioration can also reduce the richness of prey in insectivorous bat diets in disturbed habitats and the long-term impacts of this are not yet fully understood [81,82]. Similarly, Estrada-Villegas et al. [73] showed fragmentation increased the activity of aerial insectivorous forest bats and altered their assemblage composition. This is also reflected in the responses of other taxonomic groups, including birds, plants, and invertebrates [1]. Therefore, we cannot rule out the presence of edge effects by measuring activity alone. Nevertheless, our study does demonstrate that if edge effects are present, common bat species have been able to adapt their behaviour to cope with them at their current magnitude. More specialist species are less adaptable and therefore are more vulnerable to potential edge effects.

### Considerations for study design

**Fig 5:**
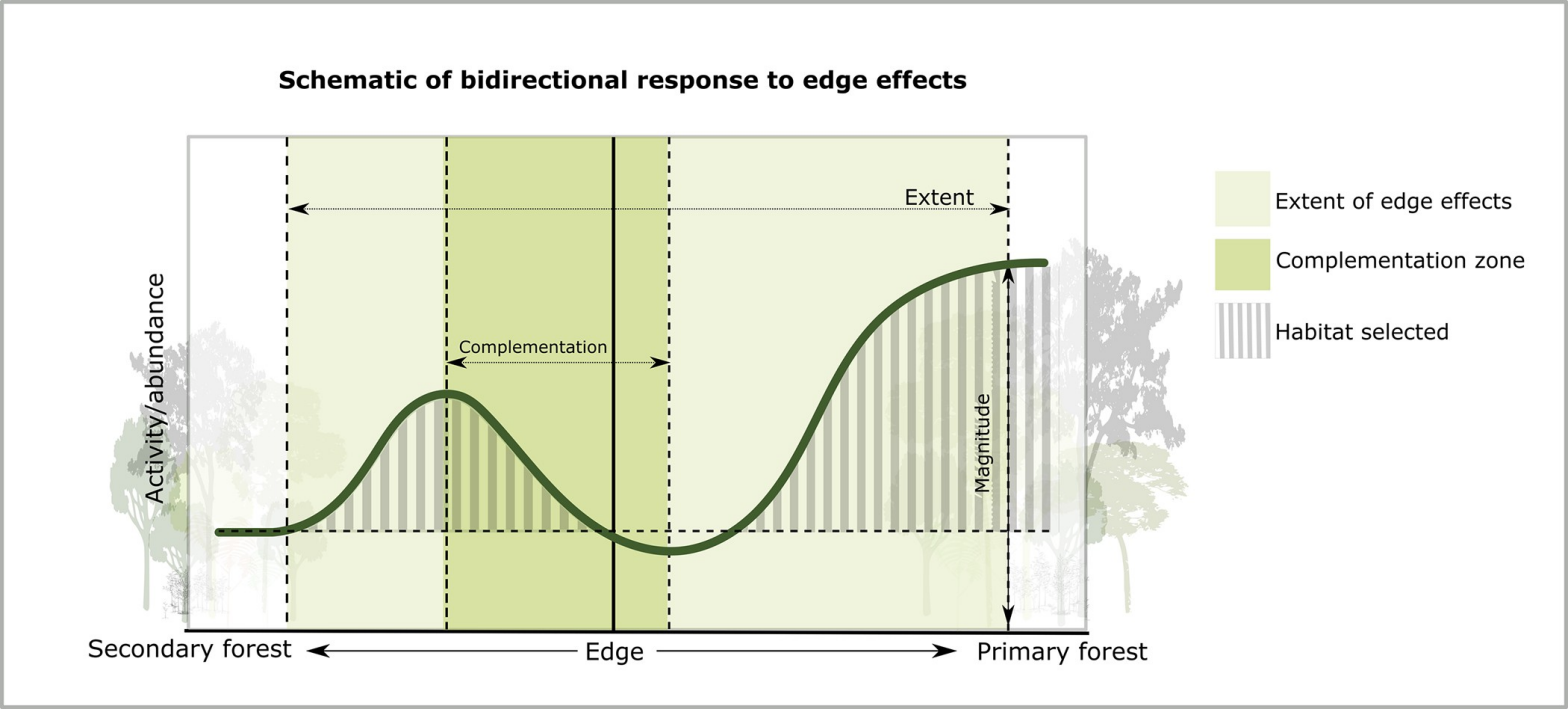
Schematic of bidirectional response to edge effects. A theoretical example of how activity/abundance may exhibit a bidirectional response to edge effects. The complementation zone would be the area between the first asymptotes from the edge in each habitat. The full extent of edge effects is observed at the second asymptotes from the edge in each habitat whereby activity stabilises.

One limitation of the statistical approach employed here is that the models by Ewers and Didham [19] assume a unidirectional response to edge effects in each habitat [19,24]. This does not necessarily account for the interaction between habitats at the border. As previously discussed, individuals may leave the primary forest to exploit resources in the secondary forest within a certain distance from the edge [29,33,83]. This may create an inflated decrease of activity in the immediate area adjacent to the edge in the primary forest (Fig 5). Habitat complementation, the use of different habitats across a landscape, is the key process thought to underpin the distribution of mobile species in heterogeneous landscapes, including bats [83]. Further studies should consider incorporating a model (e.g., a spline regression model) which could test for bidirectional responses to edge effects, e.g., where activity increases in the first 200 m from the edge but then decreases for 400 m before stabilizing to natural activity levels (Fig 5; hypothetical values). Whilst it would not be possible to calculate magnitude and extent from this type of model, it would help to test for source-sink dynamics (see [29]).

For management purposes, future research could also examine the impact that different land clearing approaches have on later regeneration. This study was predominantly restricted to *Vismia*-dominated regrowth, therefore further studies could also investigate whether comparable patterns are observed with a matrix dominated by *Cecropia* regrowth. This would enable land-owners to clear the forest in a manner (with or without the use of fire) that would minimize its effects on bat assemblages (see [69]). Additionally, we recommend future studies extend the transect length and repeat across more replicates. This will help identify the extent of edge effects for forest specialists, as well as eliminate the risk that extent is not being detected for other guilds due to sampling design. Increased replication may also facilitate species-specific analyses using Ewers and Didham’s [19] models which were not possible in this study due to small sample sizes.

Only one species classified as an edge forager demonstrated a preference for the forest edge in our study. This may be because our “edge” did not represent a hard edge between forest and non-forest. Therefore, our findings support Jantzen & Fenton [84] which suggests this type of labelling oversimplifies the relationship between species and edge effects and does not capture variation in species responses due to different types of edge. As matrix contrast plays a pivotal role in determining the impact of edge effects [12,26–28], future research would also benefit from comparing the responses we observed in a low-contrast matrix to those detected in high-contrast matrix landscapes, e.g., in soy plantations, without first classifying species into guilds.

## Conclusions

Investigating how we can buffer the impacts of edge effects will be increasingly important to protect species in human-modified tropical landscapes. Our results demonstrate that maintaining secondary forest in an advanced regeneration state (> 30 years) adjacent to primary forest can help support common aerial insectivorous bats at the landscape level. However, it also highlights that edge effect responses can be guild- and species-specific and that their increased specialization means forest specialists are more susceptible to edge effects, even in a mosaic of primary and ca. 30-year-old secondary forests. Consequently, primary forest remains irreplaceable for supporting the whole bat assemblage. We advocate that future studies also consider how vertical stratification and source-sink dynamics may affect species responses to edge effects. Whilst secondary forest in isolation may not be able to support the same bat diversity and abundance as primary forest, we argue it can reduce extinction pressure from edge effects at the landscape level and mitigate habitat degradation in the remaining primary forest. Therefore, the long-term protection of secondary forests would greatly benefit the conservation of neotropical bats in human-modified landscapes.

## Supporting information

S1 Table The training data for the classifier and the comparison between manual classification and automatic classification. The Wilcoxon test was used to compare the difference between the number of bat passes (≥ 2 pulses) automatically identified by the classifier to at least 60% confidence (Auto ID) compared to manual identification (Manual ID) in the understory. “–” represents insufficient files for statistical comparison. Training data represents the total number of individual pulses available to train the classifier, see López-Baucells et al. [58] for full methodology.
(DOCX)

S2 Table Model parameter estimates after fitting Ewers and Didham’s [19] edge effect models. Each model below represents the best-fit model(s) per guild and stratum as determined using the second-order Akaike Information Criterion (AICc). These include the raw estimates on the logarithmic scale as well as the back-transformed estimates (true bat passes). Mean number of bat passes in stratum (η or β0). Change in bat passes with distance from the edge (β1). Confidence intervals (CI) for the transformed scale were calculated using the delta method.
(DOCX)

S3 Table Generalized linear mixed-effect model equations. Generalized linear mixed-effect model (GLMMs) equations used to model bat activity (n) as a function of the distance from the forest edge (*Distance*), forest type (*ForestType*) and stratum (*Strata*) for each of the three bat guilds and per species. The models are ordered based on their AICc. **Bold**–top three models per guild.
(DOCX)

S4 Table Results of likelihood ratio tests comparing the top generalized linear mixed-effect models for each guild and species/sonotype (see S3 Table). (DOCX)
