# Supplementary material for: Edge effects and vertical stratification of aerial insectivorous bats across the interface of primary-secondary Amazonian rainforest
Source: PLoS One. 2022 Sep 23;17(9):e0274637. doi: 10.1371/journal.pone.0274637 (PMC9506665; doi:10.1371/journal.pone.0274637)
Supplement: S1 Table — The Wilcoxon test was used to compare the difference between the number of bat passes (≥ 2 pulses) automatically identified by the classifier to at least 60% confidence (Auto ID) compared to manual identification (Manual ID) in the understory. “–” represents insufficient files for statistical comparison. Training data represents the total number of individual pulses available to train the classifier, see López-Baucells et al. [58] for full methodology. (DOCX) [file pone.0274637.s001.docx]

| **S1 Table –** **The training data for the classifier and the comparison between manual classification and automatic classification.**  The Wilcoxon test was used to compare the difference between the number of bat passes  (≥ 2 pulses) automatically identified by the classifier to at least 60% confidence (Auto ID) compared to manual identification (Manual ID) in the understory. “–” represents insufficient files for statistical comparison. Training data represents the total number of individual pulses available to train the classifier, see López-Baucells et al. (2019) for full methodology. | | | | | | |
| --- | --- | --- | --- | --- | --- | --- |
| Guild | Species/sonotype | Training data | Auto ID | Manual ID | W-value | p-value |
| Forest specialist | |  |  |  |  |  |
|  | *Eptesicus brasiliensis* | 125,490 | 130 | 22 | 22 | 0.52 |
|  | *Furipterus horrens* | 1,125 | 35 | 24 | - | - |
|  | *Myotis riparius* | 1,191,184 | 533 | 521 | 471 | 0.59 |
| Flexible forest forager | |  |  |  |  |  |
|  | *Pteronotus gymnonotus* | 8,362 | 241 | 58 | 9 | 0.787 |
|  | *Pteronotus alitonus* | 506,515 | 10,295 | 10,643 | 5414 | 0.99 |
|  | *Pteronotus* cf. *rubiginosus* | 622,328 | 8,569 | 8,374 | 5203 | 1 |
| Edge forager | |  |  |  |  |  |
|  | *Cormura brevirostris* | 40,397 | 451 | 282 | 123260 | 0.87 |
|  | *Centronycteris maximiliani/centralis* | 685,227 | 15,215 | 20,386 | 3321 | 0.72 |
|  | *Peropteryx kappleri* | 149,007 | 105 | 146 | 57.5 | 0.86 |
|  | *Peropteryx macrotis* | 14,176 | 535 | 21 | - | - |
|  | *Saccopteryx bilineata* | 510,960 | 3,024 | 3565 | 1807.5 | 0.72 |
|  | *Saccopteryx leptura* | 262,826 | 922 | 1310 | 1570 | 0.73 |
|  |  |  |  |  |  |  |
| Not used for subsequent analysis | |  |  |  |  |  |
|  | *Emballonuridae spp.* | 11,068 | 369 | 2 | - | - |
|  | *Molossidae spp.* | 40,281 | 1,215 | 216 | 43995 | < 0.01 |
|  | *Pteronotus personatus* | 663 | 63 | 2 | - | - |
