# Supplementary material for: Edge effects and vertical stratification of aerial insectivorous bats across the interface of primary-secondary Amazonian rainforest
Source: PLoS One. 2022 Sep 23;17(9):e0274637. doi: 10.1371/journal.pone.0274637 (PMC9506665; doi:10.1371/journal.pone.0274637)
Supplement: S2 Table — Each model below represents the best-fit model(s) per guild and stratum as determined using the second-order Akaike Information Criterion (AICc). These include the raw estimates on the logarithmic scale as well as the back-transformed estimates (true bat passes). Mean number of bat passes in stratum (η or β0). Change in bat passes with distance from the edge (β1). Confidence intervals (CI) for the transformed scale were calculated using the delta method. (DOCX) [file pone.0274637.s002.docx]

| S2 Table – Model parameter estimates after fitting Ewers and Didham’s (2006) edge effect models.  Each model below represents the best-fit model(s) per guild and stratum as determined using the second-order Akaike Information Criterion (AICc). These include the raw estimates on the logarithmic scale as well as the back-transformed estimates (true bat passes). Mean number of bat passes in stratum (η or β0). Change in bat passes with distance from the edge (β1). Confidence intervals (CI) for the transformed scale were calculated using the delta method. | | | | | |
| --- | --- | --- | --- | --- | --- |
|  | **Stratum** | **Model** | **Parameter** | **Raw**  **estimate (CI)** | **Transformed estimate (CI)** |
| Forest specialists | |  |  |  |  |
|  | Canopy |  |  |  |  |
|  |  | Null | $\bar{\eta}$ | 2.56 (2.26-2.86) | 12.91 (9.11-16.71) |
|  | Understory |  |  |  |  |
|  |  | Linear | β_0_ | 0.957 (0.742-1.172) | 2.60  (2.06-3.14) |
|  |  | Linear | β_1_ | -0.000371 (-0.000735- -0.00000751) |  |
|  |  | Power | β_0_ | 0.937 (0.711-1.162) | 2.55  (1.99-3.11) |
|  |  | Power | β_1_ | -0.000366 (-0.000759- -0.0000270) |  |
| Flexible forest foragers | |  |  |  |  |
|  | Canopy |  |  |  |  |
|  |  | Null | $\bar{\eta}$ | 3.32  (3.03-3.62) | 27.77  (19.82-35.72) |
|  | Understory |  |  |  |  |
|  |  | Null | $\bar{\eta}$ | 3.63  (3.28-3.98) | 37.67 (24.99-50.35) |
| Edge species | |  |  |  |  |
|  | Canopy |  |  |  |  |
|  |  | Null | $\bar{\eta}$ | 5.82  (5.37-6.27) | 337.07  (189.81-484.33) |
|  | Understory |  |  |  |  |
|  |  | Null | $\bar{\eta}$ | 2.85  (2.32-3.39) | 17.36  (8.40-26.32) |
