# Supplementary material for: Edge effects and vertical stratification of aerial insectivorous bats across the interface of primary-secondary Amazonian rainforest
Source: PLoS One. 2022 Sep 23;17(9):e0274637. doi: 10.1371/journal.pone.0274637 (PMC9506665; doi:10.1371/journal.pone.0274637)
Supplement: S3 Table — Generalized linear mixed-effect model (GLMMs) equations used to model bat activity (n) as a function of the distance from the forest edge (Distance), forest type (ForestType) and stratum (Strata) for each of the three bat guilds and per species. The models are ordered based on their AICc. Bold–top three models per guild. (DOCX) [file pone.0274637.s003.docx]

| **S3 Table –** **Generalized linear mixed-effect model equations.**  Generalized linear mixed-effect model (GLMMs) equations used to model bat activity (n) as a function of the distance from the forest edge (*Distance*), forest type (*ForestType*) and stratum (*Strata*) for each of the three bat guilds and per species. The models are ordered based on their AICc. **Bold** – top three models per guild. | | | | |
| --- | --- | --- | --- | --- |
|  |  | Model | | AICc |
| **Guild** | | |  |  |
| Forest specialists | | |  |  |
|  |  | **n ~** | ***Strata* + *ForestType* + (1 \| Transect) + (1 \| Season)** | **3310.382** |
|  |  | **n ~** | ***Strata* + (1 \| Transect) + (1 \| Season)** | **3310.627** |
|  |  | **n ~** | ***Strata* + *ForestType* + *Distance* + (1 \| Transect) + (1 \| Season)** | **3311.989** |
|  |  | n ~ | ***Strata* + *Distance* + (1 \| Transect) + (1 \| Season)** | 3312.595 |
|  |  | n ~ | ***ForestType* + (1 \| Transect) + (1 \| Season)** | 3411.355 |
|  |  | n ~ | 1 + (1 \| Transect) + (1 \| Season) | 3412.537 |
|  |  | n ~ | *Distance* + (1 \| Transect) + (1 \| Season) | 3412.547 |
|  |  |  |  |  |
| Flexible forest  foragers | | |  |  |
|  |  | **n ~** | ***Strata* + *ForestType* + *Distance* + (1 \| Transect) + (1 \| Season)** | **2690.872** |
|  |  | **n ~** | ***Strata* + *ForestType* + (1 \| Transect) + (1 \| Season)** | **2692.605** |
|  |  | **n ~** | ***Strata* + *Distance* + (1 \| Transect) + (1 \| Season)** | **2692.917** |
|  |  | n ~ | *Strata* + (1 \| Transect) + (1 \| Season) | 2693.108 |
|  |  | n ~ | *ForestType* + (1 \| Transect) + (1 \| Season) | 2700.977 |
|  |  | n ~ | *Distance* + (1 \| Transect) + (1 \| Season) | 2702.108 |
|  |  | n ~ | 1 + (1 \| Transect) + (1 \| Season) | 2702.218 |
|  |  |  |  |  |
| Edge foragers | | |  |  |
|  |  | **n ~** | ***Strata* + *ForestType* + (1 \| Transect) + (1 \| Season)** | **1761.389** |
|  |  | **n ~** | ***Strata* + *ForestType* + *Distance* + (1 \| Transect) + (1 \| Season)** | **1763.509** |
|  |  | **n ~** | ***Strata* + (1 \| Transect) + (1 \| Season)** | **1771.861** |
|  |  | n ~ | ***Strata* + *Distance* + (1 \| Transect) + (1 \| Season) +** | 1773.974 |
|  |  | n ~ | *ForestType* + (1 \| Transect) + (1 \| Season) | 1851.106 |
|  |  | n ~ | 1 + (1 \| Transect) + (1 \| Season) | 1851.642 |
|  |  | n ~ | *Distance* + (1 \| Transect) + (1 \| Season) | 1853.739 |
| **Forest Specialist Species** | | |  |  |
| *Eptesicus brasiliensis* | |  |  |  |
|  | | **n ~** | ***Strata* + (1 \| Transect) + (1 \| Season)** | **987.978** |
|  | | **n ~** | ***Strata* + *Distance* + (1 \| Transect) + (1 \| Season)** | **989.711** |
|  | | **n ~** | ***Strata* + *ForestType* + (1 \| Transect) + (1 \| Season)** | **990.234** |
|  | | n ~ | *Strata* + *ForestType* + *Distance* + (1 \| Transect) +  (1 \| Season) | 990.921 |
|  | | n ~ | *ForestType* + (1 \| Transect) + (1 \| Season) | 1018.763 |
|  | | n ~ | *Distance* + (1 \| Transect) + (1 \| Season) | 1019.818 |
|  | | n ~ | 1 + (1 \| Transect) + (1 \| Season) | 1019.948 |
|  | |  |  |  |
| *Furipterus horrens* | |  |  |  |
|  | | n ~ | **1 + (1 \| Transect) + (1 \| Season)** | **146.394** |
|  | | n ~ | ***Distance* + (1 \| Transect) + (1 \| Season)** | **149.024** |
|  | | n ~ | ***Strata* + (1 \| Transect) + (1 \| Season)** | **149.162** |
|  | | n ~ | *ForestType* + (1 \| Transect) + (1 \| Season) | 151.232 |
|  | | n ~ | *Strata* + *Distance* + (1 \| Transect) + (1 \| Season) | 152.021 |
|  | | n ~ | *Strata* + *ForestType* + (1 \| Transect) + (1 \| Season) | 154.477 |
|  | | n ~ | *Strata* + *ForestType* + *Distance* + (1 \| Transect) +  (1 \| Season) | 157.920 |
|  | |  |  |  |
| *Myotis riparius* | |  |  |  |
|  |  | **n ~** | ***Strata* + *ForestType* + (1 \| Transect) + (1 \| Season)** | **1552.907** |
|  |  | **n ~** | ***Strata* + *ForestType* + *Distance* + (1 \| Transect) +  (1 \| Season)** | **1555.012** |
|  |  | **n ~** | ***Strata* + (1 \| Transect) + (1 \| Season)** | **1564.228** |
|  |  | n ~ | *Strata* + *Distance* + (1 \| Transect) + (1 \| Season) | 1566.209 |
|  |  | n ~ | *ForestType* + (1 \| Transect) + (1 \| Season) | 1609.111 |
|  |  | n ~ | 1 + (1 \| Transect) + (1 \| Season) | 1613.556 |
|  |  | n ~ | *Distance* + (1 \| Transect) + (1 \| Season) | 1615.246 |
|  |  |  |  |  |
| **Flexible Forest Foragers** | | |  |  |
| *Pteronotus gymnonotus* | |  |  |  |
|  | | **n ~** | ***Strata* + *ForestType* + (1 \| Transect) + (1 \| Season)** | **825.721** |
|  | | **n ~** | ***Strata* + *ForestType* + *Distance* + (1 \| Transect) +  (1 \| Season)** | **827.901** |
|  | | **n ~** | ***ForestType* + (1 \| Transect) + (1 \| Season)** | **829.755** |
|  | | n ~ | *Strata* + (1 \| Transect) + (1 \| Season) | 832.027 |
|  | | n ~ | *Strata* + *Distance* + (1 \| Transect) + (1 \| Season) | 834.147 |
|  | | n ~ | 1 + (1 \| Transect) + (1 \| Season) | 835.240 |
|  | | n ~ | *Distance* + (1 \| Transect) + (1 \| Season) | 837.269 |
|  | |  |  |  |
| *Pteronotus alitonus* | |  |  |  |
|  | | **n ~** | ***Distance* + (1 \| Transect) + (1 \| Season)** | **2456.393** |
|  | | **n ~** | **1 + (1 \| Transect) + (1 \| Season)** | **2456.641** |
|  | | **n ~** | ***Strata* + *Distance* + (1 \| Transect) + (1 \| Season)** | **2456.764** |
|  | | n ~ | *Strata* + (1 \| Transect) + (1 \| Season) | 2457.133 |
|  | | n ~ | *Strata* + *ForestType* + *Distance* + (1 \| Transect) +  (1 \| Season) | 2459.124 |
|  | | n ~ | *ForestType* + (1 \| Transect) + (1 \| Season) | 2459.284 |
|  | | n ~ | *Strata* + *ForestType* + (1 \| Transect) + (1 \| Season) | 2459.934 |
|  | |  |  |  |
| *Pteronotus* cf. *rubiginosus* | |  |  |  |
|  | | **n ~** | ***Strata* + *ForestType* + (1 \| Transect) + (1 \| Season)** | **1775.579** |
|  | | **n ~** | ***Strata* + (1 \| Transect) + (1 \| Season)** | **1776.131** |
|  | | **n ~** | ***Strata* + *ForestType* + *Distance* + (1 \| Transect) +  (1 \| Season)** | **1777.657** |
|  | | n ~ | *Strata* + *Distance* + (1 \| Transect) + (1 \| Season) | 1778.264 |
|  | | n ~ | *ForestType* + (1 \| Transect) + (1 \| Season) | 1792.464 |
|  | | n ~ | 1 + (1 \| Transect) + (1 \| Season) | 1792.486 |
|  | | n ~ | *Distance* + (1 \| Transect) + (1 \| Season) | 1794.505 |
|  | |  |  |  |
|  | |  |  |  |
| **Edge Foragers** | |  |  |  |
| *Cormura brevirostris* | |  |  |  |
|  | | **n ~** | ***Strata* + *ForestType* + *Distance* + (1 \| Transect) +  (1 \| Season)** | **1524.520** |
|  | | **n ~** | ***Strata* + *ForestType* + (1 \| Transect) + (1 \| Season)** | **1525.402** |
|  | | **n ~** | ***Strata* + *Distance* + (1 \| Transect) + (1 \| Season)** | **1525.542** |
|  | | n ~ | *Strata* + (1 \| Transect) + (1 \| Season) | 1527.525 |
|  | | n ~ | *ForestType* + (1 \| Transect) + (1 \| Season) | 1589.172 |
|  | | n ~ | *Distance* + (1 \| Transect) + (1 \| Season) | 1589.855 |
|  | | n ~ | 1 + (1 \| Transect) + (1 \| Season) | 1589.958 |
|  | |  |  |  |
| *Centronycteris maximiliani/ centralis* | |  |  |  |
|  | | **n ~** | ***Strata* + (1 \| Transect) + (1 \| Season)** | **2832.336** |
|  | | **n ~** | ***Strata* + *Distance* + (1 \| Transect) + (1 \| Season)** | **2833.596** |
|  | | **n ~** | ***Strata* + *ForestType* + (1 \| Transect) + (1 \| Season)** | **2836.077** |
|  | | n ~ | *Strata* + *ForestType* + *Distance* + (1 \| Transect) +  (1 \| Season) | 2836.96 |
|  | | n ~ | 1 + (1 \| Transect) + (1 \| Season) | 2882.936 |
|  | | n ~ | *Distance* + (1 \| Transect) + (1 \| Season) | 2883.696 |
|  | | n ~ | *ForestType* + (1 \| Transect) + (1 \| Season) | 2886.056 |
|  | |  |  |  |
| *Peropteryx kappleri* | |  |  |  |
|  | | **n ~** | ***Strata* + *Distance* + (1 \| Transect) + (1 \| Season)** | **1324.164** |
|  | | **n ~** | ***Strata* + (1 \| Transect) + (1 \| Season)** | **1325.739** |
|  | | **n ~** | ***Strata* + *ForestType* + *Distance* + (1 \| Transect) +  (1 \| Season)** | **1328.431** |
|  | | n ~ | *Strata* + *ForestType* + (1 \| Transect) + (1 \| Season) | 1329.548 |
|  | | n ~ | *Distance* + (1 \| Transect) + (1 \| Season) | 1378.069 |
|  | | n ~ | 1 + (1 \| Transect) + (1 \| Season) | 1380.900 |
|  | | n ~ | *ForestType* + (1 \| Transect) + (1 \| Season) | 1382.126 |
|  | |  |  |  |
| *Peropteryx macrotis* | |  |  |  |
|  | | **n ~** | ***Strata* + *Distance* + (1 \| Transect) + (1 \| Season)** | **1696.556** |
|  | | **n ~** | ***Strata* + *ForestType* + *Distance* + (1 \| Transect) +  (1 \| Season)** | **1699.403** |
|  | | **n ~** | ***Strata* + (1 \| Transect) + (1 \| Season)** | **1710.847** |
|  | | n ~ | *Strata* + *ForestType* + (1 \| Transect) + (1 \| Season) | 1713.381 |
|  | | n ~ | *Distance* + (1 \| Transect) + (1 \| Season) | 1773.567 |
|  | | n ~ | 1 + (1 \| Transect) + (1 \| Season) | 1798.948 |
|  | | n ~ | *ForestType* + (1 \| Transect) + (1 \| Season) | 1800.654 |
|  | |  |  |  |
| *Saccopteryx bilineata* | |  |  |  |
|  | | **n ~** | ***Strata* + *ForestType* + (1 \| Transect) + (1 \| Season)** | **2158.266** |
|  | | **n ~** | ***Strata* + *ForestType* + *Distance* + (1 \| Transect) +  (1 \| Season)** | **2160.289** |
|  | | **n ~** | ***Strata* + (1 \| Transect) + (1 \| Season)** | **2194.951** |
|  | | n ~ | *Strata* + *Distance* + (1 \| Transect) + (1 \| Season) | 2196.387 |
|  | | n ~ | *ForestType* + (1 \| Transect) + (1 \| Season) | 2215.687 |
|  | | n ~ | *Distance* + (1 \| Transect) + (1 \| Season) | 2243.731 |
|  | | n ~ | 1 + (1 \| Transect) + (1 \| Season) | 2246.840 |
|  | |  |  |  |
| *Saccopteryx leptura* | |  |  |  |
|  | | **n ~** | ***Strata* + *ForestType* + (1 \| Transect) + (1 \| Season)** | **1972.092** |
|  | | **n ~** | ***Strata* + *ForestType* + *Distance* + (1 \| Transect) +  (1 \| Season)** | **1974.278** |
|  | | **n ~** | ***Strata* + (1 \| Transect) + (1 \| Season)** | **1987.207** |
|  | | n ~ | *Strata* + *Distance* + (1 \| Transect) + (1 \| Season) | 1989.261 |
|  | | n ~ | *ForestType* + (1 \| Transect) + (1 \| Season) | 2087.837 |
|  | | n ~ | 1 + (1 \| Transect) + (1 \| Season) | 2097.085 |
|  | | n ~ | *Distance* + (1 \| Transect) + (1 \| Season) | 2098.459 |
