# Supplementary material for: Edge effects and vertical stratification of aerial insectivorous bats across the interface of primary-secondary Amazonian rainforest
Source: PLoS One. 2022 Sep 23;17(9):e0274637. doi: 10.1371/journal.pone.0274637 (PMC9506665; doi:10.1371/journal.pone.0274637)
Supplement: S4 Table — (DOCX) [file pone.0274637.s004.docx]

| **S4 Table – Results of likelihood ratio tests comparing the top generalized linear mixed-effect models for each guild and species/sonotype (see Table S3).** | | | | | |
| --- | --- | --- | --- | --- | --- |
|  | Covariate | Chi-squ | DF | *P* Value |  |
| Forest specialists |  |  |  |  |  |
|  | *Strata* | 91.85 | 1 | 2.2^e-16^ | *** |
|  | *ForestType* | 14.77 | 2 | 6.199^e-04^ | *** |
|  | *Distance* | 0.04 | 1 | 0.834 |  |
| Flexible forest foragers |  |  |  |  |  |
|  | *Strata* | 10.44 | 1 | 1.233^e-03^ | ** |
|  | *ForestType* | 6.32 | 2 | 0.042 | * |
|  | *Distance* | 3.88 | 1 | 0.049 | * |
| Edge foragers |  |  |  |  |  |
|  | *Strata* | 101.40 | 1 | 2.2^e-16^ | *** |
|  | *ForestType* | 4.88 | 2 | 0.087 | * |
|  | *Distance* | 0.54 | 1 | 0.462 |  |
| *Eptesicus brasiliensis* |  |  |  |  |  |
|  | *Strata* | 27.90 | 1 | 1.28^e-07^ | *** |
|  | *ForestType* | 3.25 | 2 | 0.197 |  |
|  | *Distance* | 1.56 | 1 | 0.211 |  |
| *Furipterus horrens* |  |  |  |  |  |
|  | *Strata* | 3.4^e-03^ | 1 | 0.954 |  |
|  | *ForestType* | 0.87 | 2 | 0.647 |  |
|  | *Distance* | 0.08 | 1 | 0.782 |  |
| *Myotis riparius* |  |  |  |  |  |
|  | *Strata* | 58.23 | 1 | 2.331^e-14^ | *** |
|  | *ForestType* | 15.534 | 2 | 4.235^e-04^ | *** |
|  | *Distance* | 0.08 | 1 | 0.783 |  |
| *Pteronotus gymnonotus* |  |  |  |  |  |
|  | *Strata* | 6.15 | 1 | 0.013 | * |
|  | *ForestType* | 10.68 | 2 | 0.005 | ** |
|  | *Distance* | 0.05 | 1 | 0.820 |  |
| *Pteronotus alitonus* |  |  |  |  |  |
|  | *Strata* | 1.58 | 1 | 0.209 |  |
|  | *ForestType* | 1.92 | 2 | 0.383 |  |
|  | *Distance* | 2.96 | 1 | 0.085 |  |
| *Pteronotus* cf. *rubiginosus* |  |  |  |  |  |
|  | *Strata* | 18.91 | 1 | 1.372^e-05^ | *** |
|  | *ForestType* | 5.00 | 2 | 0.083 |  |
|  | *Distance* | 1.11 | 1 | 0.736 |  |
| *Cormura brevirostris* |  |  |  |  |  |
|  | *Strata* | 67.45 | 1 | 2.22^e-16^ | *** |
|  | *ForestType* | 5.37 | 2 | 0.068 |  |
|  | *Distance* | 3.07 | 1 | 0.080 |  |
| *Centronycteris maximiliani/centralis* |  |  |  |  |  |
|  | *Strata* | 51.82 | 1 | 6.083^e-13^ | *** |
|  | *ForestType* | 0.94 | 2 | 0.625 |  |
|  | *Distance* | 1.28 | 1 | 0.258 |  |
| *Peropteryx kappleri* |  |  |  |  |  |
|  | *Strata* | 53.37 | 1 | 2.757^e-13^ | *** |
|  | *ForestType* | 0.19 | 2 | 0.908 |  |
|  | *Distance* | 3.36 | 1 | 0.067 |  |
| *Peropteryx macrotis* |  |  |  |  |  |
|  | *Strata* | 79.61 | 1 | 2.2^e-16^ | *** |
|  | *ForestType* | 1.51 | 2 | 0.470 |  |
|  | *Distance* | 16.17 | 1 | 5.79^e-05^ | *** |
| *Saccopteryx bilineata* |  |  |  |  |  |
|  | *Strata* | 57.3 | 1 | 3.741^e-14^ | *** |
|  | *ForestType* | 40.44 | 2 | 1.654^e-09^ | *** |
|  | *Distance* | 0.16 | 1 | 0.689 |  |
| *Saccopteryx leptura* |  |  |  |  |  |
|  | *Strata* | 117.87 | 1 | 2.2^e-16^ | *** |
|  | *ForestType* | 19.33 | 2 | 6.334^e-05^ | *** |
|  | *Distance* | 2^e-03^ | 1 | 0.964 |  |
